# Physiologically Guided Modeling for EEG Multichannel Signals

**DOI:** 10.3390/bioengineering13020131

**Published:** 2026-01-23

**Authors:** Christian Canedo, Cristina Rueda

**Affiliations:** Department of Statistics and Operations Research, University of Valladolid, 47002 Valladolid, Spain; cristina.rueda@uva.es

**Keywords:** signal analysis, EEG, Frequency-Modulated Möbius, dipole

## Abstract

Electroencephalographic (EEG) recordings often exhibit strong interchannel correlations due to scalp potentials reflecting electric fields generated by localized neural sources commonly modeled as current dipoles. Despite this physiological basis, many widely used approaches (e.g., independent component analysis) are largely data-driven, may require many components, and offer limited interpretability and limited support for assessing physiological assumptions. We propose a physiologically guided multichannel model built on a parametric Frequency-Modulated Möbius (FMM) formulation, where each electrode signal is expressed as a linear combination of a small number of latent dipole-related sources with parametrically described trajectories. The resulting framework captures temporal and spatial structure with high accuracy and enables likelihood-based testing of the fixed dipole-orientation assumption through a reduced-rank formulation. Using publicly available EEG data, we illustrate that the proposed approach explains interchannel dependence with few latent sources and provides statistical evidence regarding the plausibility of fixed orientation.

## 1. Introduction

EEGis a non-invasive technique that measures voltage fluctuations generated by synchronized activity of cortical pyramidal neurons using multiple electrodes placed on the scalp [1]. EEG is widely used in clinical and research settings, supporting applications such as epilepsy diagnosis [2], sleep staging [3], anesthesia monitoring [4], and the assessment of neurodegenerative and psychiatric disorders [5,6,7]. Beyond these classical clinical applications, EEG has become increasingly relevant in computational psychiatry and affective computing. In major depressive disorder (MDD), recent studies leverage advanced feature engineering, time-frequency representations, and machine/deep learning methods to improve diagnostic classification and to assess the practical feasibility and limitations of EEG-based biomarkers [8,9,10,11,12]. In parallel, EEG-based emotion recognition has grown rapidly due to its potential in human–computer interaction, with modern approaches combining signal transformations and deep neural architectures, alongside recent reviews synthesizing methodological trends and evaluation practices [13,14,15,16,17].

Physiologically, EEG activity arises when groups of pyramidal neurons become synchronously active. Their combined currents can be approximated at a large scale by an equivalent current dipole, a vector that summarizes the overall strength and direction of the neural activity within a small cortical region [18]. The voltage measured at each electrode is the result of the superposition of the fields produced by many dipoles active across the cortex. Consequently, each EEG channel reflects a mixture of these underlying contributions, forming a multivariate time series whose oscillatory patterns encode the dynamics of the neural generators [19,20,21,22,23]. Accurate modeling is essential for identifying rhythms, tracking temporal changes, and deriving clinically useful biomarkers; this calls for models that link cortical sources to the observed signals [7,21,24,25].

Most existing analytical approaches, such as independent component analysis (ICA) or principal component analysis (PCA), decompose the multichannel signal into a set of data-driven components, e.g., [26,27,28]. Although these methods have proved effective for artifact removal and exploratory analysis, they offer limited physiological interpretability and no explicit link to the dipolar generators that underlie EEG activity; moreover, recent benchmarking studies have shown that reasonable preprocessing choices can influence ERP estimates and downstream conclusions [29,30,31]. In ICA, the most popular approach, the observed channels are modeled as linear mixtures of statistically independent sources, a simple statistical representation that, however, ignores the spatial and biophysical structure. Moreover, in EEG, there is no fixed or predetermined relationship between the location of a scalp electrode and the underlying cortical source or sources that generate the recorded signals. The field of source analysis encompasses a range of methods developed to estimate how many sources are active and where they are located within the brain. However, this area remains surrounded by significant uncertainty and controversy, as these methods rely on a number of debatable assumptions. One particularly problematic assumption concerns the orientation of the dipoles representing the neural sources [20,22,32]. Despite its central role in electrophysiological modeling, there remains considerable debate over whether the orientation of cortical dipoles should be assumed fixed or allowed to vary across time and experimental conditions. To date, this assumption has rarely been examined through formal statistical testing, leaving a long-standing hypothesis in EEG analysis largely unverified [5,18,19,33].

The framework developed in this work addresses this need by providing a physiologically motivated way to represent dipolar neural generators and their temporal evolution, offering a clearer and more interpretable connection between cortical activity and multichannel EEG recordings. We propose a physiologically guided multivariate FMM model as a reduced-rank variant within the FMM framework [34,35]. The model represents scalp potentials as the superposition of a small number of latent FMM sources. Each source corresponds to a cortical dipole whose moment vector has a parametric temporal evolution and projects onto channel-specific directions, producing the observed voltages.

This additive structure reflects the physical laws of electric field propagation. Furthermore, the FMM model is fully parametric, providing a statistically efficient and interpretable representation of multichannel EEG. Its physiologically guided structure, which explicitly incorporates interchannel relationships, yields stable parameter estimation and improves robustness to noise.

Beyond signal representation, the proposed framework allows a formal statistical comparison between models assuming fixed and variable dipole orientations. By embedding this hypothesis within a likelihood-based reduced-rank formulation, we provide a unified framework for estimating dipole dynamics and formally comparing variable- and fixed-orientation models through rank constraints, within a single likelihood-based approach.

Empirical analyses on publicly available datasets demonstrate that our model yields a more parsimonious and physiologically grounded representation of oscillatory activity. This capability bridges statistical modeling and physiological validation in EEG analysis, offering a unified framework for both representation and inference.

The remainder of the paper is organized as follows. Section 2 recalls the biophysical formulation of EEG potentials in terms of dipole position and orientation, reviews the main elements of the FMM model and introduces the concept of reduced-rank multivariate modeling. Section 3 presents the proposed restricted FMM model, describing how physical constraints are incorporated, the estimation algorithm and how the model permits formal validation of the fixed-orientation hypothesis. Section 4 reports empirical analyses using publicly available EEG data, and Section 5 concludes with a discussion of implications and future research directions.

## 2. Background

### 2.1. Biophysical Formulation

The electric potentials measured on the scalp by an EEG are generated by the summed activity of large populations of cortical pyramidal neurons, which can be approximated by a set of equivalent current dipoles, e.g., [5,18,19].

The equivalent dipole moment d(t) represents the net current flow generated by a population of cortical pyramidal neurons within a localized region. Its magnitude and orientation jointly reflect the balance between excitatory and inhibitory synaptic activity and the degree of temporal synchronization among neurons. At rest, when neuronal firing is largely uncorrelated and synaptic currents are statistically balanced, the net dipole moment fluctuates near zero. Following stimulation or spontaneous synchronization, a subset of pyramidal neurons becomes transiently active, generating coherent current flow along their dendritic axes. Consequently, d(t) increases in magnitude, reaches a peak corresponding to maximal population activation, and subsequently declines as the cortical tissue returns to baseline. The temporal trajectory depends on physiological and experimental conditions.

Traditionally, EEG modeling assumes that the orientation of the dipole remains constant and only its magnitude varies in time [36]. Under this classical model, d(t) evolves linearly along a fixed direction, representing a localized and stable cortical generator whose activity is purely amplitude modulated. This assumption is supported by the near-parallel organization of pyramidal neurons in a cortical column, which results in a macroscopic current flow along a nearly fixed axis. However, real cortical activity is often more complex and spatially distributed than this idealization suggests [37,38]. Recent work indicates that dipole orientation can vary over time in ways that may reflect underlying physiological dynamics, and the combined activity of nearby cortical regions may produce gradual changes in the effective dipole direction [33]. In such situations, the trajectory of d(t) can be reasonably approximated by an elliptical path within a plane, representing the smooth evolution of the resultant dipole orientation. This generalized formulation does not imply a physical rotation of a single current element, but rather provides a compact geometrical description of distributed or anisotropic cortical activation.

Distinguishing between linear and elliptical trajectories of d(t) thus provides a methodological means to evaluate the adequacy of the source model and to characterize differences in the spatial organization of cortical activity across conditions.

### 2.2. Geometric Representation

Let R denote the vector from the source to the channel, and let R=∥R∥ be its length. The scalar potential V(t) generated by an electric dipole in a homogeneous, infinite, and isotropic medium of conductivity σ is given by

(1) $$V\left(t\right)=\frac{1}{4\pi \sigma }\;\frac{\langle d(t),R\rangle }{R^{3}}$$

The dipole vector d(t) does not represent a position but a directed quantity (the dipole moment).

We assume that each dipole is described by an elliptical trajectory lying in an arbitrary plane. An elliptical trajectory provides a natural geometric description of a dipole that is not confined to a single direction and is therefore adopted as a flexible geometric model of dipolar dynamics. Under this assumption, the dipole evolves along an ellipse contained in a plane $\Pi \subset R^{3}$. Consider an orthonormal basis $\left\{e_{1},e_{2},e_{3}\right\}$ adapted to that plane, where $e_{1},e_{2}\in \Pi$. The trajectory of the dipole can be written as

$$d\left(t\right)=L_{1}\cos\phi \left(t\right)\;e_{1}+L_{2}\sin\phi \left(t\right)\;e_{2}+z\;e_{3}.$$

**Figure 1:**
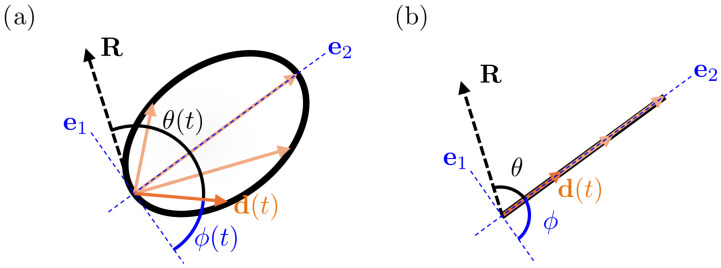
Geometricrepresentation of the dipole-based model. In both panels, several orange arrows illustrate the evolution of the dipole moment along its trajectory over time; a single representative instance is explicitly labeled as d(t). In black, R denotes the source–channel direction, and θ(t) is the instantaneous angle between d(t) and R. (**a**) Elliptical (variable-orientation) evolution: the dipole moment moves without reversals on an ellipse lying in a plane $\Pi \subset R^{3}$, described in an adapted orthonormal basis $\left\{e_{1},e_{2},e_{3}\right\}$ with $e_{1},e_{2}\in \Pi$. The phase ϕ(t) (shown in blue) parametrizes the position along the trajectory. (**b**) Degenerate linear (fixed-orientation) case: when one semi-axis vanishes (e.g., $L_{1}=0$), the elliptical trajectory collapses to a one-dimensional subspace, corresponding to a constant dipole orientation with time-varying magnitude.

Importantly, the phase ϕ(t) is an internal parameter of the dipole trajectory and should not be confused with the angle θ(t) which denotes the instantaneous angle between d(t) and the source–channel direction R; see Figure 1a.

A purely linear trajectory arises as a degenerate case of this parametrization: when one of the semi-axes vanishes and, without loss of generality, we assume $L_{1}=0$, the ellipse collapses into a line segment; see Figure 1b. The same phase function still provides a valid representation, although the resulting curve no longer explores a two-dimensional elliptical geometry.

From now on, the superscript *d* denotes quantities associated with dipole *d*, and the subscript *g* denotes quantities associated with channel *g*. For each dipole–channel pair, let $R_{g}^{d}\in R^{3}$ denote the corresponding source–channel direction.

The scalar product of the dipole moment with this direction can be written as

(2) $$\langle d^{d}\left(t\right),R_{g}^{d}\rangle =L_{1}^{d}\cos\left(\phi^{d}\left(t\right)\right)\;\langle e_{1}^{d},R_{g}^{d}\rangle +L_{2}^{d}\sin\left(\phi^{d}\left(t\right)\right)\;\langle e_{2}^{d},R_{g}^{d}\rangle +z^{d}\;\langle e_{3}^{d},R_{g}^{d}\rangle .$$

Therefore, in the ideal homogeneous infinite-medium case, the contribution of dipole *d* to the signal measured at channel *g*, denoted by $Y_{g}^{d}\left(t\right)$, is given by $\langle d^{d}\left(t\right),R_{g}^{d}\rangle$ up to a time-independent constant $C_{g}^{d}$.

Using the identity Acos(ϕ)+Bsin(ϕ)=Ccos(ϕ−η), $Y_{g}^{d}\left(t\right)$ can be rewritten as

(3) $$Y_{g}^{d}\left(t\right)=m_{g}^{d}\;+\;A_{g}^{d}\cos\left(\phi^{d}\left(t\right)+\eta_{g}^{d}\right),$$

where

$$m_{g}^{d}=z^{d}C_{g}^{d}\;\langle e_{3}^{d},R_{g}^{d}\rangle ,$$

and the amplitude $A_{g}^{d}$ and phase offset $\eta_{g}^{d}$ are given by

(4) $$A_{g}^{d}=C_{g}^{d}\sqrt{{\left(L_{1}^{d}\langle e_{1}^{d},R_{g}^{d}\rangle \right)}^{2}+{\left(L_{2}^{d}\langle e_{2}^{d},R_{g}^{d}\rangle \right)}^{2}},\;\eta_{g}^{d}=-atan2\left(L_{2}^{d}\langle e_{2}^{d},R_{g}^{d}\rangle ,\;L_{1}^{d}\langle e_{1}^{d},R_{g}^{d}\rangle \right).$$

Finally, since the recorded voltage at channel *g* is the superposition of the contributions from *D* dipoles, the observation model takes the form

(5) $$Y_{g}\left(t\right)=M_{g}\;+\;\sum_{d=1}^{D}A_{g}^{d}\cos\left(\phi^{d}\left(t\right)+\eta_{g}^{d}\right)\;+\;\varepsilon_{g}\left(t\right),$$

where $M_{g}=\sum_{d=1}^{D}m_{g}^{d}$ and $\varepsilon_{g}\left(t\right)∼N\left(0,\sigma_{g}^{2}\right)$ represents additive Gaussian noise.

### 2.3. FMM Representation

The dipole is assumed to traverse its trajectory without reversals. Since the motion is fully determined by the phase function ϕ(t), this assumption requires ϕ(t) to be monotonic. From a modeling perspective, explicitly parametrizing ϕ(t) is advantageous, as it yields a small and interpretable set of parameters and promotes parsimony. Such parsimony is essential because the number of active dipoles is rarely known a priori; without structural assumptions of this kind, the EEG inverse problem becomes severely under-determined and non-unique [22,39].

The simplest monotonic choice is a linear phase, ϕ(t)=ωt, corresponding to uniform angular motion. A slightly richer alternative is provided by Möbius-type reparameterizations, which preserve monotonicity while allowing for non-uniform traversal of the ellipse. These functions represent the simplest nonlinear extensions compatible with circular geometry and are defined as

(6) $$\phi \left(t\right)=\beta +2\;\arctan\left(\;\omega \;\tan\left(\frac{t-\alpha }{2}\right)\right).$$

This phase function is used to describe the temporal trajectory of the dipole vector. The parameter α sets the temporal shift and thus the latency of the response, ω controls how fast the trajectory is traversed around its maximum, and β determines the relative timing of the rising and falling phases of the dipole magnitude.

The projection of an elliptical trajectory therefore yields a unimodal but potentially asymmetric waveform, as a direct geometric consequence, whose skewness depends on the geometry of the ellipse and the projection direction. Waveforms based on this parametrization are known as FMM modulations. Previous studies [34,35] have shown that FMM models provide a compact and flexible parametric representation for oscillatory signals.

In multichannel settings such as EEG, where data on multiple electrodes are recorded, the observed signals arise as linear mixtures of the same dipole sources. Each electrode records the projection of the same elliptical trajectory onto its own direction. Accordingly, all channels are assumed to share the same parameters $\left(\alpha_{d},\omega_{d},\beta_{d}\right)$ governing the instantaneous phase $\phi_{d}\left(t\right)$ of each dipole source, while electrode-specific differences are captured through $A_{g}^{d}$ and $\eta_{g}^{d}$ parameters. Multichannel FMM representations have been successfully applied to multivariate time series, including ECG and EEG, enabling accurate modeling of frequency variation and waveform asymmetry [40,41].

### 2.4. Reduced-Rank Regression

In the classical multivariate regression model

(7) Y=XB+E,

where Y is an n×p matrix of response variables, X is an n×q matrix of predictors, B is a q×p matrix of regression coefficients, and E denotes the n×p matrix of errors, a reduced-rank constraint is imposed by requiring that

rank(B)=r<min(q,p).

This assumption implies that the responses depend on a limited number of latent linear combinations of the predictors. Equivalently, the coefficient matrix can be factorized as

(8) $$B=GH^{⊤},$$

where G is a q×r matrix and H is a p×r matrix. The model can thus be written as

(9) $$Y=XGH^{⊤}+E,$$

so that the *p* response variables are explained through *r* latent predictor combinations XG.

Estimation of G and H is based on minimizing the sum of squared residuals

(10) $$\min_{G,\;H}\mathrm{tr}\left[{\left(Y-XGH^{⊤}\right)}^{⊤}\left(Y-XGH^{⊤}\right)\right],\;\mathrm{subject}\;\mathrm{to}\;\mathrm{rank}\left(GH^{⊤}\right)=r.$$

The least-squares solution is obtained from the singular value decomposition (SVD) of the sample cross-covariance between predictors and responses, which provides the best rank-*r* approximation to the multivariate regression surface in the least-squares sense [42,43]. The singular vectors associated with the largest *r* canonical correlations define the subspaces that maximize the explained cross-covariance between X and Y, establishing a close connection between reduced-rank regression and canonical correlation analysis.

## 3. Proposed Model

Let $\left\{t_{1},\ldots ,t_{T}\right\}\subset \left[0,2\pi \right]$ denote the sampling points, with $0\leq t_{1}<\cdots <t_{T}\leq 2\pi$ and $\vartheta =(\alpha_{1},\omega_{1},\ldots ,\alpha_{D},\omega_{D})$ collect the nonlinear parameters of the *D* sources. Using the reparametrization $\delta_{g}^{d}:=\eta_{g}^{d}+\beta^{d}$, for each $t\in \{t_{1},\ldots ,t_{T}\}$, models (5) and (6) can be written as

(11) $$Y\left(t\right)=M+B\;H\left(\vartheta ,t\right)+\varepsilon \left(t\right),\;\varepsilon \left(t\right)∼N_{G}\left(0,\Sigma \right),$$

where $Y\left(t\right)\in R^{G}$ is the vector of voltages across channels, $M\in R^{G}$ is the channel-wise baseline, $B\in R^{G\times 2D}$ is the spatial mixing matrix with

(12) $$B_{g,2d-1}=A_{g}^{d}\cos\left(\delta_{g}^{d}\right),\;B_{g,2d}=-A_{g}^{d}\sin\left(\delta_{g}^{d}\right),$$

and $H\left(\vartheta ,t\right)\in R^{2D}$ collects the temporal regressors

(13) $$H_{2d-1}\left(\vartheta ,t\right)=\cos\left(\psi^{d}\left(t\right)\right),\;H_{2d}\left(\vartheta ,t\right)=\sin\left(\psi^{d}\left(t\right)\right),$$

where $\psi^{d}\left(t\right)=2\;\arctan\left(\;\omega^{d}\tan\left(\frac{t-\alpha^{d}}{2}\right)\right)$; and $\Sigma \in R^{G\times G}$ is variance-covariance matrix of the residuals.

### 3.1. Reduced-Rank Model Representing the Fixed-Orientation Hypothesis

The restriction of fixed dipole orientations translates into linear constraints on the structure of the coefficient matrix B. Going back to the geometric representation given in (3) and (4), a linear trajectory corresponds to $L_{1}^{d}=0$ which implies:

(14) $$A_{g}^{d}=\left|C_{g}^{d}L_{2}^{d}\;\langle e_{2}^{d},R_{g}^{d}\rangle \right|,\;\eta_{g}^{d}=-atan2\left(L_{2}^{d}\;\langle e_{2}^{d},R_{g}^{d}\rangle ,\;0\right)=-\frac{\pi }{2}\;\mathrm{sgn}\left(L_{2}^{d}\;\langle e_{2}^{d},R_{g}^{d}\rangle \right).$$

Therefore, when the dipole direction does not change over time, the phase shift $\eta_{g}^{d}$ collapses to $\pm \frac{\pi }{2}$. The effect of adding or subtracting $\frac{\pi }{2}$ to the argument of a cosine or a sine is given by the identities

(15) $$\cos\left(\delta_{g}^{d}\right)=\cos\left(\beta^{d}\pm \frac{\pi }{2}\right)=\mp \;\sin\left(\beta^{d}\right),\;\sin\left(\beta^{d}\pm \frac{\pi }{2}\right)=\pm \;\cos\left(\beta^{d}\right).$$

Thus, a cosine shifted by $\pm \frac{\pi }{2}$ becomes a sine, and a sine shifted by $\pm \frac{\pi }{2}$ becomes a cosine. Therefore, in the linear case the relative combination of the cosine and sine components is identical for every channel: only a single multiplicative factor changes from channel to channel.

To make this constraint explicit, let $B^{\left(d\right)}\in R^{G\times 2}$ denote the two-column block of the mixing matrix associated with dipole *d*, i.e., $B^{\left(d\right)}=\left[\;b_{2d-1}\;\;b_{2d}\;\right]$. Under the general (elliptical) formulation, $B^{\left(d\right)}$ is complete and typically has rank two. Under the fixed-orientation hypothesis, the cosine and sine spatial patterns must be proportional across channels, which is equivalently expressed as the rank-one restriction

(16) $$\mathrm{rank}\left(B^{\left(d\right)}\right)=1,\;d=1,\ldots ,D.$$

That is, there exist $u^{\left(d\right)}\in R^{G}$ and $v^{\left(d\right)}\in R^{2}$ such that $B^{\left(d\right)}=u^{\left(d\right)}{\left(v^{\left(d\right)}\right)}^{⊤}$. This reduced-rank structure provides an explicit statistical encoding of the fixed-orientation assumption at the channel level. As a consequence, the general representation with 2G channel-specific coefficients reduces to G+1 parameters: a single common temporal waveform for dipole *d*, together with one scalar multiplier per channel.

Hence, the classical fixed-orientation model is obtained as a reduced-rank special case of the general elliptical formulation. Testing these rank conditions provides a principled way to assess whether each cortical source is directionally stable (fixed) or dynamically evolving (elliptical).

### 3.2. Comparison with PCA, ICA, and Related Decomposition Methods

Classical decomposition methods such as PCA or ICA also represent multichannel signals as linear mixtures of latent components:

(17) Y(t)=BH(t)+ε(t),

where H(t) denotes a vector of latent time courses. PCA enforces orthogonality among the trajectories H(t), whereas ICA imposes statistical independence. Neither method specifies a particular temporal structure for H(t), nor assigns biophysical meaning to the columns of B.

The proposed FMM formulation (11) preserves the same linear mixing architecture but introduces two key extensions.

First, the latent trajectories are replaced by analytic FMM waveforms whose parameters determine latency, asymmetry, and frequency modulation. Thus, the complete vectors $H_{t}$ of PCA/ICA are replaced by smooth, parametric, and physiologically interpretable temporal dynamics.

Second, the coefficient matrix B acquires biophysical structure: each dipole contributes two columns corresponding to its cosine and sine projection patterns across channels. Linear relations between these columns encode hypotheses about dipole orientation. A fixed-orientation dipole leads to a rank-one column pair, whereas an elliptical dipole requires a full rank-two pair.

As a result, the FMM model is not a purely algebraic factorization but a reduced-rank nonlinear least-squares framework that integrates structured temporal dynamics with physiological spatial constraints. This yields a representation that is parsimonious, interpretable, and well-suited for EEG analysis and complementary to classical PCA/ICA.

While reduced-rank regression is not a recent technique, it continues to be relevant for analyzing multichannel neural data, including EEG, where it enables low-dimensional representations of inter-signal dependencies [44].

### 3.3. Parameter Estimation Algorithm

The parameters (B,ϑ,Σ) are estimated by maximum likelihood under the Gaussian model (11). Although the likelihood is nonlinear in ϑ, it is quadratic in B for fixed (ϑ,Σ). Therefore, for any given (ϑ,Σ), the optimal coefficient matrix $\hat{B}\left(\vartheta ,\Sigma \right)$ is available in closed form via generalized least squares, possibly under rank constraints. This naturally motivates a profile-likelihood strategy in which B is updated analytically within the optimization over ϑ.

Alternating Updates

At each iteration, the following steps are performed:

1. Update of the nonlinear parameters ϑ. Given Σ, we maximize the *profile* log-likelihood
  (18) $$\ell_{p}\left(\vartheta ;\Sigma \right):=\ell \left(\hat{B}\left(\vartheta ,\Sigma \right),\vartheta ,\Sigma \right),$$
  where $\hat{B}\left(\vartheta ,\Sigma \right)$ denotes the closed-form GLS (or reduced-rank GLS) estimate of B for the current value of ϑ. In practice, this step follows multivariate procedures described in the FMM literature, e.g., [34,35], exploiting the smoothness of the Möbius phase functions and using a profile approach with respect to $\left(\alpha_{d},\omega_{d}\right)$.
2. Update of the coefficient matrix B. Conditional on (ϑ,Σ), the model is linear in B,
  (19) Y(t)=M+BH(ϑ,t)+ε(t),
  so we set $B\leftarrow \hat{B}\left(\vartheta ,\Sigma \right)$. When orientation constraints are imposed, they are enforced at this stage through the corresponding reduced-rank least-squares update.
3. Update of Σ. The noise covariance is re-estimated from the current residuals. Under diagonal or structured assumptions this reduces to simple variance estimators; under a full covariance model it corresponds to the empirical covariance of the GLS residuals.

#### 3.3.1. Computational Properties

The alternating scheme exploits the separable structure of the likelihood: the nonlinear parameters ϑ enter only through the temporal functions, while B and Σ enter linearly. This yields stable iterations and avoids the need for joint optimization in a high-dimensional nonconvex space. In practice, only a small number of outer iterations is required for convergence, as documented in existing multivariate FMM applications [35,40].

#### 3.3.2. Initialization

The noise covariance Σ must be initialized. Following standard practice in likelihood- based multivariate models, we use as starting point the empirical covariance matrix of the observed voltages. This choice provides a consistent and data-driven estimate of the overall noise scale while ensuring that the diagonal entries of Σ are sufficiently large at initialization. In particular, setting the initial variances to values not smaller than their empirical counterparts prevents numerical degeneracies in the first iterations and guarantees that the profile-likelihood updates of the nonlinear parameters are well conditioned. The initial Σ is subsequently refined in step 3 of the algorithm using the residuals of the current FMM fit.

#### 3.3.3. Handling the Linear (Degenerate) Case

The same algorithm accommodates both the general elliptical model and the fixed-orientation (rank-one) case. The difference lies only in step 2: for fixed orientation, the two columns of B associated with each dipole are constrained to be proportional, whereas the general elliptical case allows a full two-dimensional subspace. The estimation framework thus naturally incorporates hypothesis testing on dipole orientation through rank constraints on B.

This estimation strategy yields a computationally efficient and statistically robust procedure that exploits the analytic structure of FMM waveforms while respecting the biophysical constraints of dipolar EEG generators.

#### 3.3.4. Model Comparison

Although the fixed- and variable-orientation models are nested, formal likelihood-based tests are not well suited to the present setting. In high-density EEG, both formulations involve a large number of parameters, leading to very large log-likelihood values that are highly sensitive to channel-wise noise. As a result, differences in likelihood may primarily reflect high dimensionality rather than meaningful differences in model adequacy. For similar reasons, information criteria based on the likelihood are also difficult to interpret in this context. We therefore rely primarily on descriptive goodness-of-fit measures, focusing on the coefficient of determination.

For each channel *g*, the coefficient of determination is defined as

(20) $$R_{g}^{2}\;=\;1-\frac{\sum_{t=1}^{T}{\left(Y_{g}\left(t\right)-{\hat{Y}}_{g}\left(t\right)\right)}^{2}}{\sum_{t=1}^{T}{\left(Y_{g}\left(t\right)-{\bar{Y}}_{g}\right)}^{2}},\;{\bar{Y}}_{g}=\frac{1}{T}\sum_{t=1}^{T}Y_{g}\left(t\right),$$

where ${\hat{Y}}_{g}\left(t\right)$ denotes the model-based fitted value for channel *g*.

To prevent noise-dominated channels from driving likelihood differences and covariance estimation, we examine the empirical distribution of channel-wise $R^{2}$ values obtained under the complete model and restrict model comparison to channels in its upper tail, defined here by the 95th percentile. This selection is used solely for stability and interpretability and does not constitute a population-level inferential procedure. Throughout the paper, Model A refers to the variable-orientation (complete) formulation in (11), whereas Model B denotes the fixed-orientation formulation under the rank-one restriction (16).

## 4. Empirical Evidence: Real EEG Data

### 4.1. Data and Preprocessing

We analyze a publicly available high-density EEG dataset acquired during a visual stimulation task in healthy young adults [45]. The data consist of raw EEG recordings organized according to the EEG-BIDS specification and were collected under ethical approval from the CIUSSS de l’Estrie—CHUS.

In this dataset, visual stimulation produces reproducible and spatially structured EEG responses across trials. Decomposing the EEG into latent components is therefore appropriate for capturing stimulus-related patterns while reducing the dimensionality of the high-density recordings. From a modeling standpoint, we adopt a unified representation in which all spatially coherent contributions to the signal are described within the same parametric framework.

In the context of visually evoked EEG responses, the dominant neural activity is assumed to arise from synchronized cortical populations. Over the time window of the evoked response, the resulting effective dipole is therefore expected to exhibit a relatively stable orientation. We accordingly compare a complete multivariate FMM model with a fixed-orientation variant to assess the impact of imposing this constraint on the component decomposition.

#### 4.1.1. Epoch Averaging and Stimulus Selection

For each participant and stimulus condition, EEG epochs were averaged to obtain event-related potentials (ERPs), thereby improving the signal-to-noise ratio and stabilizing the spatial structure of the responses.

Nevertheless, even after averaging, some participant–stimulus ERPs may remain dominated by noise, particularly when the evoked response is weak, temporally misaligned across trials, or based on a limited number of valid epochs. Trial-to-trial latency variability can lead to partial cancellation of stimulus-locked activity, resulting in attenuated or noisy averaged signals [46]. Such responses are not expected to be informative for hypotheses concerning the spatial organization or orientation of neural generators.

Accordingly, the present analysis focuses on participant–stimulus conditions exhibiting a reliable evoked response, as the goal is to enable a stable and meaningful comparison between competing model formulations rather than to characterize all recorded EEG signals. To implement this selection in an objective manner, the complete FMM model was fitted to each averaged ERP using a fixed number of components (K=3), and the coefficient of determination ($R^{2}$) was computed independently for each channel. Fixing *K* ensures comparable model complexity across formulations and isolates the effect of the orientation constraint.

Five participants (IDs 3, 9, 13, 19, and 30) were excluded prior to downstream analyses due to pervasive EEG signal-quality failures. Across stimulus conditions, the majority of channels were either near-flat (almost null) or dominated by white-noise–like activity, with only a very small subset, typically fewer than five channels, showing any structured variation. Under these circumstances, it was not possible to ensure that apparent activity reflected genuine stimulus-evoked voltages rather than residual noise or recording artifacts; consequently, these participants were removed from the study.

#### 4.1.2. Qualitative Illustration of Model Fit

To complement the quantitative comparison, we consider a representative participant–stimulus pair retained after the data-selection procedure. The corresponding ERP exhibits clearly structured activity, making it suitable for illustrating both goodness-of-fit and interpretability.

**Figure 2:**
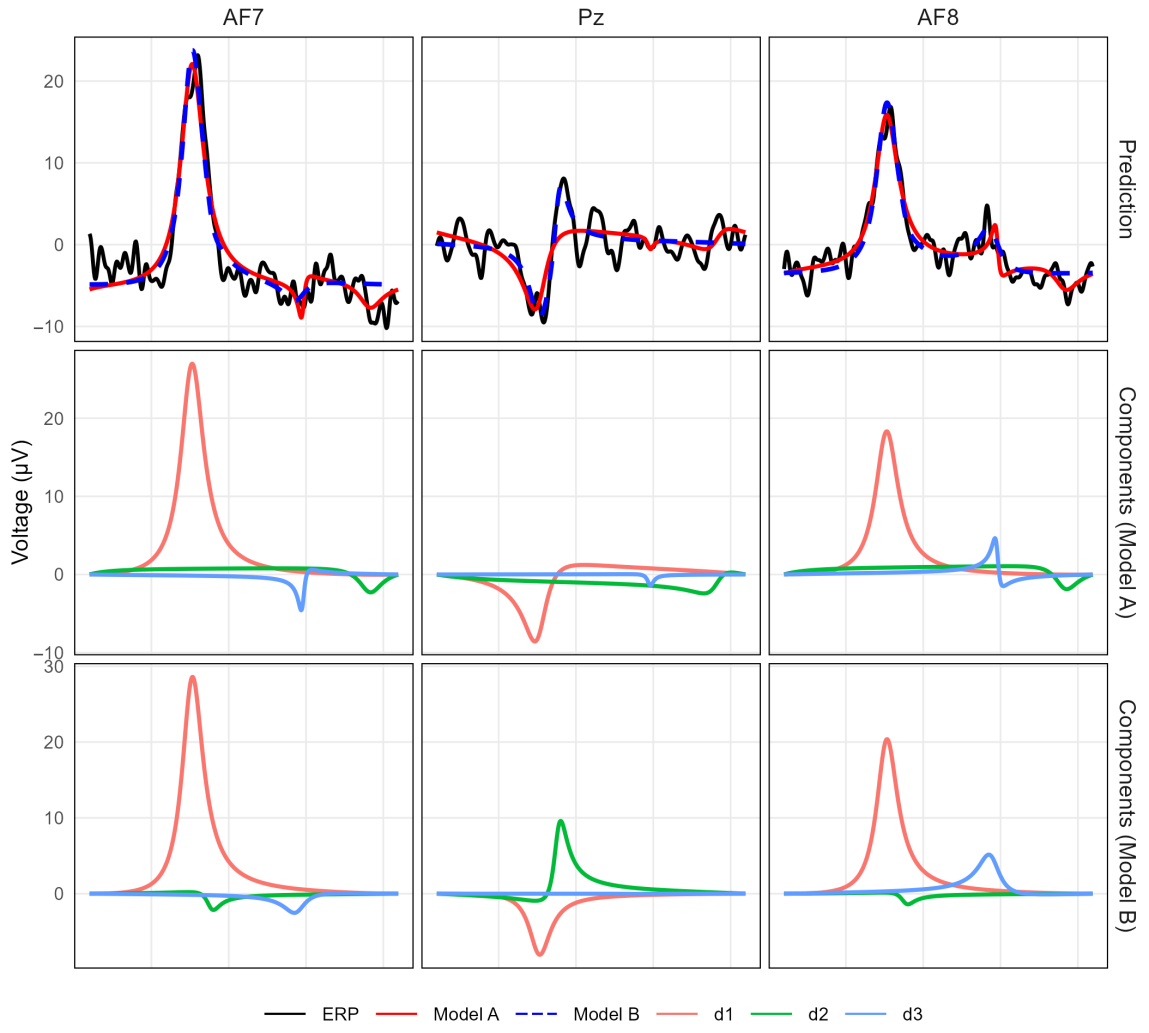
Waveform-level illustration of model fit on a representative participant-stimulus ERP for a subset of informative channels. The panels show AF7 and AF8 (left and right frontal sites) and Pz (midline posterior site). (**Top**) observed ERP together with the fitted signals from Model A and Model B. (**Middle**, **Bottom**) component time courses (d1–d3) estimated under Model A and Model B, respectively.

**Figure 3:**
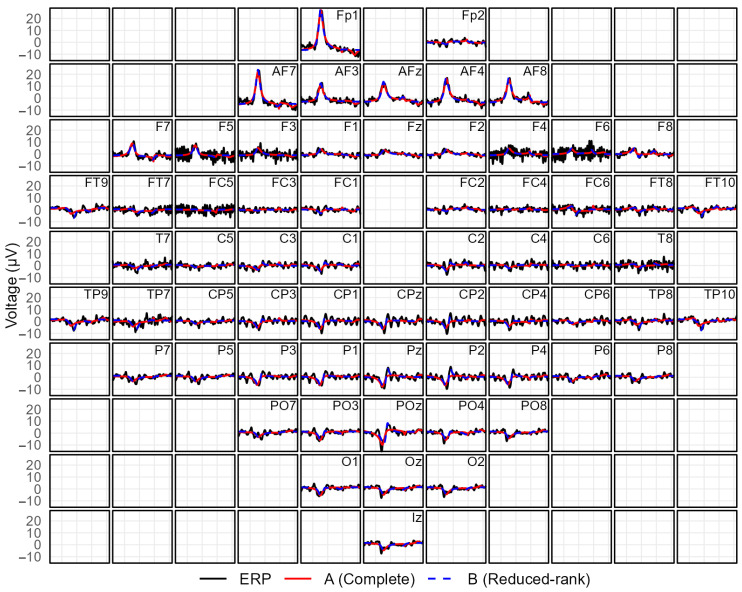

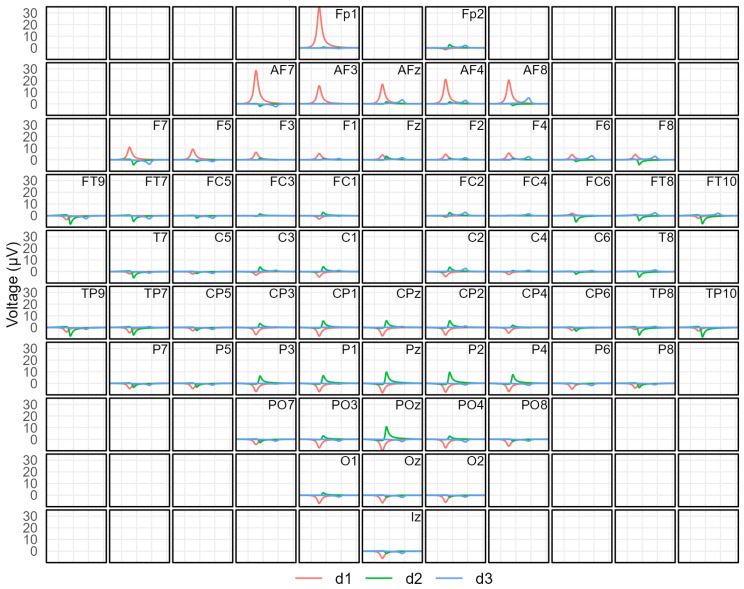
Illustration of the model fit for a representative participant and stimulus condition retained after data selection. (**Top**) Observed (black) and fitted ERP signals from the free (red) and restricted (blue) models across electrodes arranged according to their spatial location on the scalp, highlighting the ability of the model to capture the spatial organization of the evoked response. (**Bottom**) Decomposition of the fitted signal into latent restricted FMM components in a multichannel representation. The first component is dominant over frontal electrodes and shows an opposite-polarity pattern over parietal sites, whereas the second and third components appear to be more spatially concentrated over occipital and right temporal regions, respectively.

Figure 2 shows a subset of informative channels for waveform-level inspection. At waveform level, both models reconstruct the main temporal features of the ERP with comparable accuracy. Figure 3 extends the comparison to the full montage. Model A may yield components with mixed polarity across distant regions, whereas Model B tends to produce components with more spatially consistent polarity patterns. This visualization highlights that the model preserves the global spatial organization of the response while representing the multichannel ERP with a small number of structured components.

**Figure 4:**
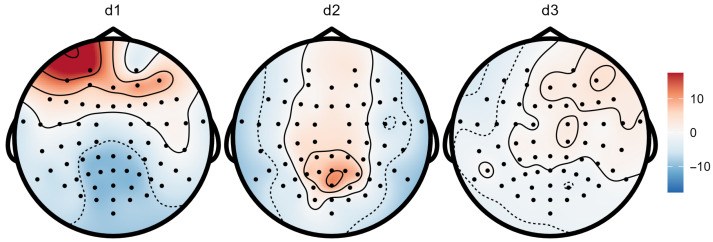
Scalp topographies of the fitted components for the reduced-rank model. For each component (**d1**–**d3**), dots indicate electrode locations (standard 10–10 layout) and colors show the interpolated spatial pattern of the component’s signed amplitude.

Finally, Figure 4 shows scalp topographic maps derived from the fitted parameters. For each component *k*, we map the channel-wise amplitude parameter $A_{g}^{k}$ from Equation (5). Under the reduced-rank model, the amplitude can take either of two opposite polarities, according to the sign of $\cos(\eta_{g}^{k})$. The resulting signed amplitudes are interpolated over the scalp using template 10–10 electrode coordinates, since subject-specific sensor locations are not available. The resulting model-based topographies provide a compact spatial summary of each latent component, highlight distinct spatial patterns across components, and offer an additional qualitative check that complements the waveform-level fits in Figure 2 and Figure 3.

### 4.2. Validation of the Hypothesis of Fixed-Orientation

The fixed-orientation hypothesis is formulated at the level of the underlying neural generators. In the context of ERPs, it is implicitly assumed that the dominant generators contributing to the averaged response are consistently engaged across trials, so that their spatial configuration is preserved under epoch averaging. Under this standard assumption, testing the fixed- versus variable-orientation formulations on averaged ERPs provides a meaningful proxy for assessing the orientation stability of the underlying dipolar sources.

Specifically, we use ERP data to compare two formulations of the proposed model: (A) a variable-orientation (complete) model and (B) fixed-orientation (reduced-rank) model.

Both models are fitted to the same participant–stimulus data using an identical number of components (K=3), thereby matching model complexity and isolating the effect of the orientation constraint. Table 1 summarizes the 95th percentile of the $R^{2}$ distribution across channels for both models.

Across participants, both model formulations provide a good description of the ERP data on the selected channels, with 95th-percentile $R^{2}$ values typically exceeding 0.80. Differences in goodness of fit between the complete and fixed-orientation models are generally small, as reflected by the near-zero average $\Delta R^{2}$, and vary in sign across individuals. In some participants, the complete model yields slightly higher $R^{2}$ values, whereas in others the fixed-orientation formulation performs comparably or better.

## 5. Discussion

The empirical results obtained from the visual evoked EEG data indicate that both the complete and the fixed-orientation formulations provide an accurate description of the dominant stimulus-locked activity across participants. The similarity in high-percentile $R^{2}$ values suggests that, for this experimental paradigm, enforcing a fixed dipole orientation provides a parsimonious and interpretable representation of the underlying dynamics. While the complete model captures additional variability in some cases, this added flexibility is not systematically required and may reflect noise in some cases rather than physiologically meaningful signal structure. These findings are consistent with the general observation that increased model complexity does not necessarily translate into improved interpretability or robustness [5,47]. In this context, the proposed framework differs from standard data-driven approaches such as ICA or PCA by explicitly incorporating physiological constraints, allowing orientation-related assumptions to be examined within the model rather than inferred post hoc.

An important aspect of the proposed approach concerns how model complexity is distributed. Instead of increasing the complexity of individual components, expressiveness is controlled through the number of simple, interpretable components. This reflects a common trade-off in EEG analysis, where similar explanatory power may be achieved using either a few complex sources or multiple simpler ones. Favoring the latter supports physiological plausibility while maintaining sufficient flexibility to describe multichannel EEG data.

Uncertainty in EEG recordings represents a key limitation when interpreting the results. EEG signals are affected by noise and artifacts arising from ocular activity, muscle contractions, and environmental factors. Although standard preprocessing steps are applied prior to model estimation, residual artifacts may still influence the fitted parameters. While the proposed framework is not designed as a dedicated artifact-removal method, its emphasis on spatially coherent activity patterns naturally attenuates unstructured noise, which may contribute to the stability of the extracted components.

## 6. Conclusions

This work introduced a statistically coherent and physiologically motivated framework for modeling multichannel EEG signals using a parametric description of cortical dipolar activity. The proposed FMM-based representation is grounded in biophysical principles and provides interpretable parameters related to latency, temporal asymmetry, and dynamical evolution of stimulus-locked responses.

Using a unified likelihood-based approach, we assessed fixed versus variable dipole-orientation formulations on visual evoked EEG data. Both formulations captured the dominant stimulus-locked activity across participants, and the fixed-orientation specification achieved comparable explanatory performance, supporting its use as a parsimonious and interpretable choice for this paradigm.

More broadly, the framework emphasizes interpretable, physiology-constrained parametrizations as an alternative to purely data-driven dimensionality reduction, and it enables coherent parameter-based spatial summaries of multichannel ERP structure.

Finally, the present study is restricted to a single paradigm and dataset and does not include a systematic quantitative comparison with alternative methods. Future work will extend the analysis to additional experimental settings and incorporate more systematic benchmarking and uncertainty quantification. Another natural continuation, motivated by the fact that the reduced-rank formulation captures the main physiologically plausible features in this setting, is to use it as an explicit ingredient in solving the EEG inverse problem. In particular, the reduced-rank multichannel structure provides a principled bridge to dipole fitting, where the estimated component-wise spatial patterns and temporal parameterizations can inform or constrain the estimation of dipole locations and orientations.

## Figures and Tables

**Table 1:** Mean $R^{2}$ values for models A and B at the 95th percentile, and their difference ($\mathrm{dif}R^{2}$) computed per participant. Model A corresponds to the variable-orientation formulation, and Model B to the fixed-orientation formulation.

Participant	$R_{A,p95}^{2}$	$R_{B,p95}^{2}$	$\mathrm{dif}R^{2}$
1	0.92121	0.91043	0.01078
2	0.80057	0.81403	−0.01346
4	0.89138	0.84567	0.04570
5	0.79337	0.75863	0.03474
6	0.90856	0.87141	0.03715
7	0.92340	0.90599	0.01741
8	0.93639	0.95328	−0.01689
10	0.88732	0.92721	−0.03989
11	0.87280	0.87551	−0.00271
12	0.85294	0.84348	0.00946
14	0.92628	0.93512	−0.00883
15	0.81764	0.79053	0.02711
16	0.87607	0.86314	0.01293
17	0.91329	0.90465	0.00864
18	0.91873	0.96464	−0.04591
20	0.85635	0.84248	0.01387
21	0.86214	0.89940	−0.03726
22	0.86566	0.89024	−0.02459
23	0.88199	0.90425	−0.02225
24	0.89479	0.90496	−0.01017
25	0.84894	0.87096	−0.02202
26	0.89798	0.88680	0.01118
27	0.77392	0.89827	−0.12434
28	0.66425	0.64146	0.02279
29	0.82653	0.88260	−0.05606
31	0.82029	0.89407	−0.07378
Total	0.86280	0.87228	−0.00948

## Data Availability

The EEG data analyzed in this study are publicly available and were obtained from the OpenNeuro repository. The dataset consists of high-density EEG recordings collected during a visual stimulation task and is accessible at https://openneuro.org [45].
